# Identification of Suicidal Ideation in the *Canadian Community Health Survey—Mental Health Component* Using Deep Learning

**DOI:** 10.3389/frai.2021.561528

**Published:** 2021-06-24

**Authors:** Sneha Desai, Myriam Tanguay-Sela, David Benrimoh, Robert Fratila, Eleanor Brown, Kelly Perlman, Ann John, Marcos DelPozo-Banos, Nancy Low, Sonia Israel, Lisa Palladini, Gustavo Turecki

**Affiliations:** ^1^ Department of Computer Science, University of Toronto, Toronto, ON, Canada; ^2^ Aifred Health Inc., Montreal, QC, Canada; ^3^ Montreal Neurological Institute, McGill University, Montreal, QC, Canada; ^4^ Department of Psychiatry, McGill University, Montreal, QC, Canada; ^5^ Faculty of Medicine, McGill University, Montreal, QC, Canada; ^6^ Department of Psychological and Brain Sciences, Boston University, Boston, MA, United States; ^7^ Douglas Mental Health University Institute, Montrea, QC, Canada; ^8^ Swansea University, Swansea, United Kingdom

**Keywords:** deep learning, suicidal ideation, risk assessment, predictors, machine learning, artificial intelligence, Canadian community health survey, Canadian community health survey—mental health 2012

## Abstract

**Introduction:** Suicidal ideation (SI) is prevalent in the general population, and is a risk factor for suicide. Predicting which patients are likely to have SI remains challenging. Deep Learning (DL) may be a useful tool in this context, as it can be used to find patterns in complex, heterogeneous, and incomplete datasets. An automated screening system for SI could help prompt clinicians to be more attentive to patients at risk for suicide.

**Methods:** Using the Canadian Community Health Survey—Mental Health Component, we trained a DL model based on 23,859 survey responses to classify patients with and without SI. Models were created to classify both lifetime SI and SI over the last 12 months. From 582 possible parameters we produced 96- and 21-feature versions of the models. Models were trained using an undersampling procedure that balanced the training set between SI and non-SI; validation was done on held-out data.

**Results:** For lifetime SI, the 96 feature model had an Area under the receiver operating curve (AUC) of 0.79 and the 21 feature model had an AUC of 0.77. For SI in the last 12 months the 96 feature model had an AUC of 0.71 and the 21 feature model had an AUC of 0.68. In addition, sensitivity analyses demonstrated feature relationships in line with existing literature.

**Discussion:** Although further study is required to ensure clinical relevance and sample generalizability, this study is an initial proof of concept for the use of DL to improve identification of SI. Sensitivity analyses can help improve the interpretability of DL models. This kind of model would help start conversations with patients which could lead to improved care and a reduction in suicidal behavior.

## Introduction

Suicide is one of the leading causes of death across the world, accounting for approximately 800,000 deaths each year, with the number of attempts an order of magnitude higher [World Health Organization (WHO), 2018]. Globally, suicide accounts for 16% of injury deaths [World Health Organization (WHO), 2012] and is the second leading cause of death in young people aged 15–29 years [World Health Organization (WHO), 2014]. This makes suicide prevention a major public health concern (Turecki and Brent, 2016). According to a meta-analysis of 365 studies, among the most important risk factors for suicide attempts and deaths are previous self-injurious behaviors and suicidal ideation (Franklin et al., 2017). Suicidal ideation includes any thoughts about suicide such as a desire for or planning of a suicide attempt and must be distinguished from actual suicidal attempts which involve acting on these thoughts (Beck et al., 1979). This is addressed by item 9 of the depression module of the Patient Health Questionnaire (PHQ-9) as “thoughts that you would be better off dead or of hurting yourself in some way” (Kroenke and Spitzer, 2002). Importantly, there is a moderately strong association between suicide and suicidal ideation, making it an important factor to consider when assessing suicide risk (Hubers et al., 2016; McHugh et al., 2019). It is important to note that this association is heterogeneous and has low positive predictive value and sensitivity (McHugh et al., 2019). As such, it is clear that not all individuals who die by suicide will have previously expressed suicidal ideation. On the other hand, suicidal ideation is much more common than attempts, and many patients who express suicidal ideation do not actually attempt suicide (Srivastava and Kumar, 2005). Regardless, proactive detection of ideation is helpful in the identification of patients at risk of suicide.

In current clinical practice, the primary method for identifying the presence of suicidal ideation is through direct questioning or patient self-report. Suicidal ideation can also be identified and characterized using instruments, such as the PHQ-9 (Kroenke et al., 2001). This method is limited because patients may conceal suicidal intentions from clinicians, who often fail to even ask about suicidal ideation (Bongiovi-Garcia et al., 2009). It would therefore be clinically useful to identify which patients may be at risk of suicidal ideation without needing to ask them directly, perhaps by using an automated screening system incorporated into the electronic medical record, as this would allow clinicians to identify patients who might benefit from further assessment and resources.

In the current literature, the vast majority of studies focus on identifying individual predictors or an interaction of only a few factors, resulting in small effect sizes with low predictive value (Franklin et al., 2017). As such, it may be useful to employ more sophisticated methods that can consider a large number of factors when making classifications. Machine learning, which allows for the creation of models that can consider many factors and identify complex relationships between them, may be an ideal tool for identifying people with suicidal ideation. While a few machine learning models have been created to predict suicide attempts or suicidal behavior (Barak-Corren et al., 2016; Passos et al., 2016; Walsh et al., 2017; DelPozo-Banos et al., 2018), we found only two that aimed at predicting suicidal ideation: Jordan et al. (2018), which focused on predicting suicidal ideation in primary care using PHQ-9 items, and Ryu et al. (2018) which demonstrated a suicidal ideation prediction model on a matched SI/non-SI sample derived from general population data. To our knowledge, we are the first group to create a model for suicidal ideation in a realistic general population, and to determine if sensitivity analyses could assist in improving the interpretability of this model.

Our objective was to train a model to identify suicidal ideation in the general population in order to include potential suicide victims who would not seek medical attention prior to their suicide attempt or who have infrequent contact with clinicians. With this goal in mind, we chose to use a deep learning model for a number of reasons. Firstly, deep learning models can be robust to missing data (Cai et al., 2018), which is common in clinical datasets. More importantly, these models are designed to find complex, non-linear patterns in data without requiring the user to specify mediators or moderators, allowing for a better approximation of the intricate relationships between the multitude of variables that put an individual at risk for suicidal thoughts.

Ideally, our model would be paired with a clinical decision support system (CDSS) that alerts clinicians and other healthcare practitioners to patients who may require further assessment and monitoring of possible suicidal thoughts. Such a tool would connect patients with their clinicians, allowing patients to fill out requested questionnaires and track their progress, while providing clinicians with an organized interface to follow the profiles of their individual patients. Similar tools have been found to be clinically useful in detecting and reducing sepsis mortality, and predicting oral cancer recurrence (Exarchos et al., 2012; Manaktala and Claypool, 2017).

Additionally, we hoped to use our machine learning approach to elucidate which patient characteristics are involved in determining the risk for suicidal ideation. This is important from a clinical perspective for understanding the factors that might cause suicidal ideation in an individual person. It is also valuable from a public health perspective, as we may discover risk factors for suicidal ideation amenable to intervention via social programs. Our approach is novel in that our sensitivity analysis allows for the identification of potential risk and protective factors.

## Materials and Methods

### Dataset

The Canadian Community Health Survey—Mental Health Component is a publicly available database provided by Statistics Canada. We therefore had no direct interactions with any patients for our study. We received ethics approval to analyze the data from the Douglas Mental Health University Institute Research Ethics Board (IUSMD-17-39). Data was collected in 2012 cross-sectionally for 25,113 people of ages 15 and over living in the ten provinces of Canada. The data was collected either by telephone or in person and 582 data points were collected per respondent. Participants were asked whether they had experienced suicidal ideation in their lifetime and in the last 12 months. The questions about SI were asked during an interview; see Box 1 for details. The response options were “yes”, “no”, “not applicable”, “don’t know”, “refusal” and “not stated”. As such, no information was provided regarding the frequency or precise timing of the suicidal ideation. We attempted to correctly identify participant answers to the lifetime and last 12 months questions separately. We included only subjects who gave a firm “yes” or “no” to the questions about suicidal ideation to maximize the discriminative ability of our model. This reduced our sample size for the identification of lifetime suicidal ideation to 23,859 with 21,597 responding “no” and 2,262 responding “yes” and the sample size for the 12 months suicidal ideation identification to 3,441 with 2,512 responding “no” and 929 responding “yes”. The size and makeup of both these subsets of the data are summarized in Supplementary Table S6. There were 485 people who responded “yes” to both questions.

Box 1 **Reproduced here from the CCHS interview guide are the specific questions asked by the interviewer about lifetime and last-12-months suicidal ideation:**

•LIFETIME
Has EXPERIENCE A (You seriously thought about committing suicide or taking your own life) ever happened to you?
•LAST 12 MONTHS
In the past 12 months, did EXPERIENCE A (You seriously thought about committing suicide or taking your own life) happen to you?

### Models

The neural network used was a feed-forward fully-connected network with three hidden layers of 400 neurons each activated by the scaled exponential linear unit (SELU) function (Klambauer et al., 2017). SELU activation paired with AlphaDropout at a 50% dropout rate for each of the hidden layers (Klambauer et al., 2017) maintains a self-normalizing property of the trained parameters of the network so as to keep the training procedure stable. Adam optimization (Kingma and Ba, 2015) with a learning rate of 1e^−4^ was used to train the network’s loss function configured as categorical cross entropy. The final prediction layer had a softmax activation, allowing the network to establish its prediction in the form of a probability for both output classes. Different neural network architectures were tried and results are presented in Supplementary Figure S3 of our Supplementary Materials. Performance decrease is apparent when the network has less than two layers or more than three layers. Thus, we chose a middle ground of three hidden layers of 400 neurons each for the rest of our analysis.

As baseline models for comparison, we include results from random forest and gradient boosting models. The random forest classifier (Breiman, 2001) was configured with 100 estimators (i.e., composed of 100 decision trees) and used the Gini impurity entropy calculation to determine the decision boundaries. It was implemented from the Scikit-learn Python package (Pedregosa et al., 2011) using all the default parameters except for those specified earlier. The gradient boosting classifier was configured with the default configurations of the Scikit-learn Python package (Pedregosa et al., 2011).

### Approach

In order to obtain a model that could be implemented in a real clinical environment, reducing the number of input features to pinpoint the most important features in the dataset is necessary. A model requiring too many input features would present challenges for data collection in the clinic when, making it difficult to apply the model to a given patient rapidly and efficiently^1^. The techniques used for feature selection involved both expertise in the field (i.e., expert feature reduction) and allowing the model to highlight which features were the most important (Guyon and Elisseeff, 2003). A clinician (D. B.) went through all 582 features and discarded the features which were either administrative (i.e., redundant case identification codes or different ways of asking the same question) or which were not reasonable to collect clinically (such as detailed health care service satisfaction metrics which would not be appropriate in a screening context where the patient has not yet experienced services fully). This reduced the feature set size to 196. We further reduced the number of features using machine learning techniques. This involved analyzing the weights fields of the trained model’s first layer and removing “unimportant” features. Feature “importance” was defined *via* the weights that the neural network applied to a particular feature, which was inspired by the concept of receptive fields in convolutional networks (Coates and Ng, 2011). Our motivation for looking at the first layer weights is that if the model effectively “drops out” that feature by masking it with a near-zero value, it will not play a role in further nonlinear interactions. This assumption can only be made for the first layer since any following intermediate layer contains too many complex interactions, precluding the association of a direct weight to a feature. We cannot make the converse assumption that high values at the first layer will equate to high feature importance because of nonlinear interactions which occur at intermediate layers. A visualization of this feature selection technique is shown in Supplementary Figure S2.

Two cases were examined, one in which 100 features were removed, leaving 96 features in the model, and one in which 175 features were removed, leaving 21 features in the model. We chose to remove 100 and 175 features respectively, since the 100 feature removal didn’t affect the performance too much from the larger feature set sizes (>100 features) and stopped at 175 because removing any more features would cause the performance to deteriorate. The larger models were produced in order to maximize the identification of important features and to maximize model accuracy; the smaller models were produced in order to generate clinically tractable models with few enough questions that they could be integrated into a standard screening assessment. Separate models were produced for both lifetime and last-12-months suicidal ideation identification.

In order to adjust our model to the large class imbalance that existed between the “no” and “yes” responders, we used undersampling. The number of examples in the majority (“no”) class was equated to the number in the minority (“yes”) class. In the case of lifetime identification, 2,262 random examples from the “no” class were randomly chosen for the training set to match the 2,262 samples from the “yes” class. The class-balanced training set was then divided into 10 different random folds, and the model was trained on nine of these folds, leaving the final fold and all of the other 19,355 “no”s to serve as the validation set. This process was repeated 10 times with mutually exclusive validation and training sets, and we noted the average of the test metrics of all runs on the validation set. It is important to mention here that our validation set was comprised of a relatively lower count of respondents in the “yes” class compared to the initial distribution of the data, making it much harder for the model to be able to classify respondents in the “yes” class correctly. The same sort of division was performed for the last-12-months data using the data distribution shown in Supplementary Table S6.

**FIGURE 1:**
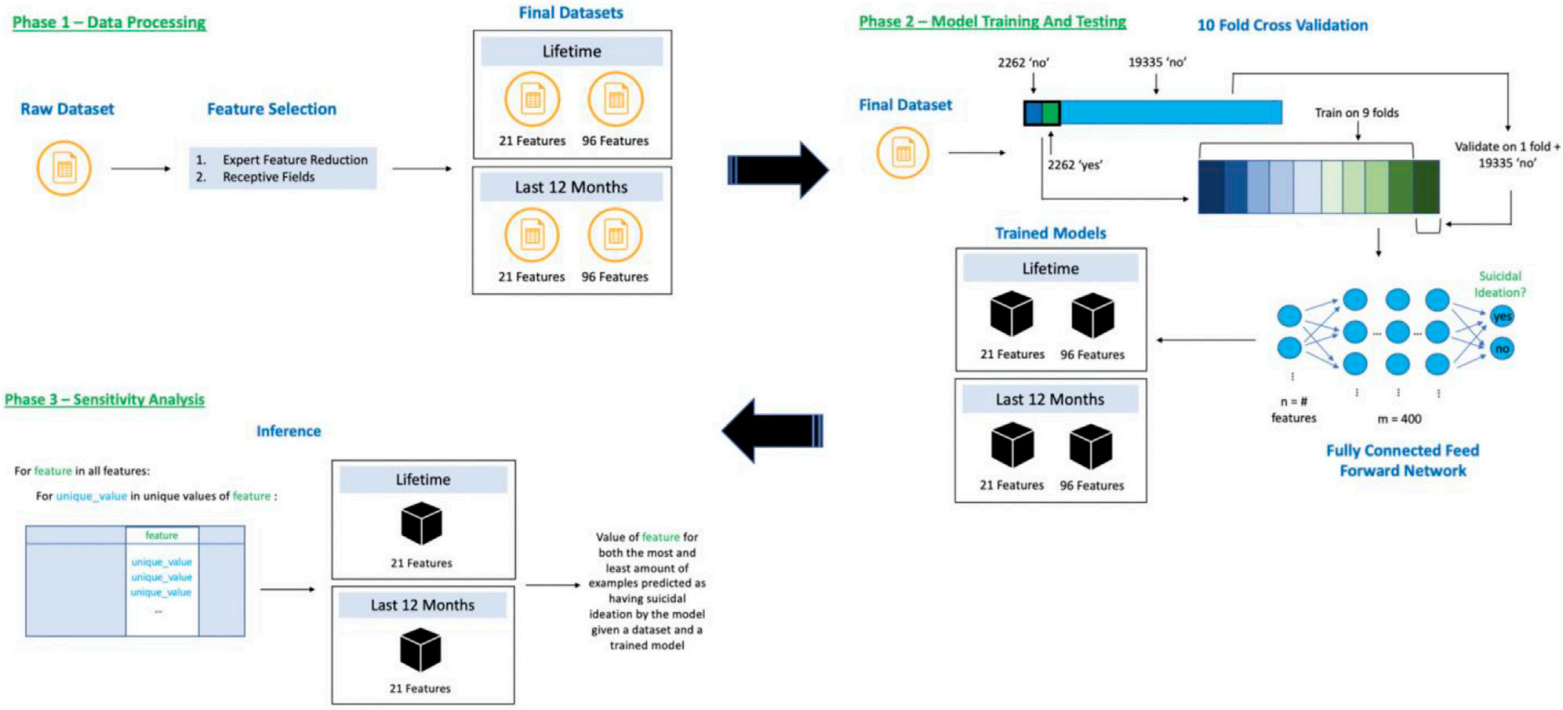
Flow of data through our training and inference system broken into three phases, 1) Data Processing—reduce dataset features using expert reduction and first layer weight analyses, 2) Model Training and Testing—10-fold cross validation using undersampling of the “no” class and training a neural network, and 3) Sensitivity Analysis—discovering feature directionality for our 21-feature trained models.

All analyses were done using the Vulcan software package (see software note). Figure 1 represents the steps taken to produce the results for this analysis.

## Results

Supplementary Appendix Tables A1–A4 show the features used for the identification of lifetime suicidal ideation (Supplementary Appendix Table A1: 96 features, Supplementary Appendix Table A2: 21 features) and suicidal ideation during the past 12 months (Supplementary Appendix Table A3: 96 features, Supplementary Appendix Table A4: 21 features). These features are those that remain following expert feature reduction (manual feature removal using domain expertise) and using the network’s first layer weights to remove additional features until 96 and 21 features remained for both lifetime and last-12-months suicidal ideation models. In terms of measurement, we chose to use the Area under the receiver operating curve (AUC) as our main metric of model performance, and we also calculated the sensitivity, specificity, negative predictive value (NPV) and positive predictive value (PPV) for each model. Supplementary Appendix Table A5 shows the 10-fold cross validated results for the lifetime (96 features—0.7890 AUC; 21 features—0.7681 AUC) and last-12-months (96 features—0.7081 AUC; 21 features—0.6798 AUC) datasets, respectively. Random forest and gradient boosted tree classifiers were produced as non-deep learning baselines; these generally performed quite well (Supplementary Appendix Table A5), with gradient boosted trees having the best metrics across all four models and random forest doing worse than deep learning in the lifetime models, but better in the last-12-months models; deep learning suffers as the training set size decreases and the last-12-months dataset is much smaller than the lifetime dataset (Supplementary Table S6). Note that the purpose of this paper was not to demonstrate superiority of deep learning, but rather to establish its feasibility and potential interpretability in this type of dataset, with the intention of using deep learning in larger datasets where, as we discuss below, it is likely to outperform other model types. In total, we produced four model configurations: 96 and 21 features for identification of lifetime suicidal ideation and 96 and 21 features for identification of suicidal ideation in the last 12 months.

In order to gain insight into how different features affected model classifications (i.e., feature directionality) and improve interpretability, we performed a feature sensitivity analysis for the 21-feature models. We chose not to perform the same analysis for the 96-feature models as it would be unsuitable to interpret due to size. We explored how variations in values for a specific feature affected the final model classification. We accomplished this by iterating through all possible unique values (up to a maximum of 20 values) for each feature and imputed all response samples to have this value. We then ran a test to determine how many of the samples would be classified as having suicidal ideation by the model. The third columns in the 21 feature tables (Supplementary Appendix Tables A2, A4) show the value of the feature where the model predicted the highest amount of suicidal ideation followed by the feature value with the lowest amount of suicidal ideation. In Supplementary Appendix Tables A2, A4, the number in brackets next to each feature value shows the number of examples in the test set classified as having suicidal ideation (19,788 samples in the test set for lifetime; 1,769 for the past 12 months). This allows for some insight into the inner workings of the neural network model. For example, in the lifetime identification of suicidal ideation, if all the answers to the question “have people to count on in an emergency” are set to “strongly disagree”, then 8,046 people are identified as having suicidal ideation; this number drops to 5,158 people if the answers are all changed to “strongly agree”.

In order to investigate model performance in subgroups which may be relevant to the prediction of suicidal ideation, we ran each of the four models three times on different splits of data, each time examining the AUCs achieved in each of a number of subgroups (these subgroups were defined by gender, household income, and education). The results can be seen in Supplementary Table S9.

## Discussion

This initial proof of concept illustrates that using our method, data from the general population can help identify people at risk of suicidal ideation. These people might benefit from more in-depth screening and resources in the context of suicide prevention, and further work using these kinds of methods might contribute to the development of clinically useful screening tools.

Jordan et al. (2018) found that using only four items of the PHQ-9 provided the most accurate predictions of suicidal ideation in their patient sample—those assessing “feelings of depression/hopelessness, low self-esteem, worrying, and severe sleep disturbances” (Jordan et al., 2018). Although the PHQ-9 was not included in our dataset, our model similarly found some high impact variables related to depression and worrying. For instance, having generalized anxiety disorder or depression were associated with suicidal ideation in our model (Supplementary Appendix Tables A1, A2). Unlike the Jordan model, ours did not identify sleep problems to be a significant risk factor for suicidal ideation. One possible explanation reconciling our results and those in the literature is that sleep problems may act as a proxy for actual interacting risk factors rather than being a risk factor themselves. When such factors are included in the data and processed by a complex model, sleep disorder factors are rendered irrelevant. We will seek to verify this hypothesis in other datasets with more robust measures of sleep. While Jordan’s model identified low self-esteem as a risk factor, our dataset unfortunately did not contain a self-esteem variable. Our model yielded additional predictive factors that do not overlap with those found by the Jordan team. Generalized anxiety disorder, for example, appears to be an important predictor of suicidal ideation (Supplementary Appendix Tables A1–A3). This is to be expected, since previous research has identified anxiety disorders, including generalized anxiety disorder, as independently predictive of suicidal ideation (Sareen et al., 2005; Bentley et al., 2016). Importantly, our method yielded predictors related to early traumatic experiences and diagnosis of PTSD. Non-consensual sexual experiences before the age of 16, appear to be associated with suicidal ideation, as is early physical abuse and number of types of childhood trauma experienced (Supplementary Appendix Tables A1–A4). This finding is supported by previous research linking increased suicidal ideation and suicide attempts to early sexual abuse, and early physical abuse to suicidal ideation through an association with anxiety, which was also an important factor identified by the model (Basile et al., 2006; Ullman et al., 2009; Bedi et al., 2011; Lopez-Castroman et al., 2013; Bahk et al., 2017; Thompson et al., 2018), thus confirming our model’s capacity to identify known risk factors of suicidal ideation. There is extensive literature suggesting that early-life adversity is an important predictor of suicidal behavior (Brezo et al., 2008; Wanner et al., 2012; Turecki and Brent, 2016). In fact, adverse childhood experiences were demonstrated to account for 67% of the population attributable risk for suicide attempts (Teicher et al., 2016).

It is interesting to note that some factors, such as generalized anxiety disorder in the lifetime 21-features model, do not have a clear directionality (i.e., they have a ratio in the sensitivity analysis close to one), and yet are included in the model as important risk or protective factors. This may be because these factors interact with other factors to produce their effect. For example in the same model physical abuse in childhood was strongly associated with SI. This opens the possibility that we are replicating the results reported by Bahk et al. (2017), where childhood physical abuse was predictive of suicidal ideation through an association with anxiety.

An important aspect of our model is potential for generalizability to a real population. We note that another paper, by Ryu et al. (2018), built a random forest classifier for the identification of suicidal ideation in a general population sample. Their approach was to downsample their data to achieve a 1:1 ratio between those endorsing and not endorsing suicidal ideation, even in their test set. Our work improves on this approach as our validation fold included a significant, more realistic, class imbalance with those not reporting suicidal ideation vastly outnumbering those who did and our results being representative of how such a model would perform when applied to a population where a class imbalance exists. As such, our model may be more generalizable to a real population.

Like ours, the Ryu model (2018) found depression and anxiety to be some of the most important features predicting SI. Sociodemographic features such as age, sex, education and features related to quality of life and employment were also predictive of SI in both models. Medical comorbidities were predictive in both models, however Ryu also noted that somatic symptoms predicted SI.

We separated identification of suicidal ideation occuring in the last 12 months and throughout the lifetime to disambiguate more specific short-term from long-term predictors. Identification of protective factors and risk factors for both conditions may improve methods of identifying and treating those at risk of attempting suicide. Lifetime factors may be useful in developing more long-term suicide prevention strategies, while factors identifying suicide ideation in the last 12 months can inform the identification and treatment of patients at more immediate risk. While all predictors were related to physical and mental wellbeing, mental health, early abusive experiences, socioeconomic situation and social support, some differences between risk factors and protective factors for the lifetime and last 12 months conditions may be important to consider. For example, having a trustworthy person to turn to for advice, seems to have significant weight in the last 12 months model. This may indicate that measures of social support, specifically of close support relationships, could be used to identify patients at more immediate risk of suicidal ideation. Based on previous literature, lack of social support may be a moderator between life stress and suicidal ideation, suggesting that a strong social support system may be beneficial in reducing suicidal thoughts, particularly during stressful times (Yang and Clum, 1994; Vanderhorst and McLaren, 2005); this seems to be relevant to both the last-12-months and lifetime models.

Additionally, contact with the police was present in the expanded models and may be associated with suicidal ideation, highlighting a need to follow up with people who may have had a traumatic experience leading to police intervention, or negative interactions with the police (DeVylder et al., 2018).

We identified several predictors that are easy to obtain, including sociodemographic features. Interestingly, Jordan et al. did not find sociodemographic features useful in the prediction of suicidal ideation (2018), but as we were using a census dataset with a large and varied array of sociodemographic features, we were able to identify more predictors amongst them that would be amenable to upstream intervention. As opposed to more expensive data like neuroimaging and genetic testing, sociodemographic predictors can be very useful in clinical practice, especially with respect to screening, since they are easily accessible to healthcare professionals through direct questioning or self-report questionnaires.

As can be seen in Supplementary Appendix Table A5, the 96-feature models have higher AUCs. This is to be expected, as the network is able to make better identifications when it has more information on the different patients it is ingesting. It is worthwhile to discuss the pros and cons of having larger or smaller models. Large models that do not overfit allow us to identify more predictors, which may be modifiable and are therefore potentially useful from a public health standpoint. Smaller models are easier to implement because patients need to answer fewer questions in order to provide the model with sufficient information to make a prediction. Thus, there exists an interesting trade-off between model accuracy and ease of data acquisition when selecting the number of features to include. For example, the difference in the AUC for the last-12-months model presented here is 0.68 for the 21-feature model vs. 0.71 for the 96-feature model. Does this difference justify a larger model that is more accurate but more difficult to collect? while the difference between the two AUC values may seem insignificant, when considering predictions on a population scale we might expect a significant difference in the absolute number of people correctly classified. Implementation of models such as these will hinge on finding the right balance between model complexity and accuracy in order to provide models that are both meaningful and feasible to implement.

It is also important to note the high negative predictive values (NPV) of our predictions. This metric indicates that the network is almost always correct when it classifies an example as not having suicidal ideation. This is crucial, as it signifies one potential use of our model may be in helping, alongside good clinical judgement and history taking, to rule out suicidal ideation in populations matching those in the dataset. However, it should be noted that given the rarity of suicidal ideations in this population, a high NPV is to be expected given that “not suicidal” is the dominant prediction class (Belsher et al., 2019)]. Given that clinicians currently have difficulty ruling out suicidal thinking or risk (McDowell et al., 2011), such a tool could eventually be clinically helpful if the rate of false negatives is judged to be acceptable. Further research is required to determine an acceptable false negative rate and how to better integrate these kinds of models into clinical decision making. Concerns about false negatives must be balanced against the risk of false positives, which can lead to unnecessary intervention and confinement, as well as against the fact that the absence of suicidal ideation at a single point in time does not rule out the risk of suicide (McHugh et al., 2019). However, given that this model identifies suicidal ideation and not risk of attempt, a positive result could be used to open a conversation between a clinician and patient, which might lead to more appropriate assessment and treatment before the risk of an attempt increases. This in turn may become a useful approach for the prevention of suicide *via* upstream identification of at-risk patients in the general population, though this remains speculative and should be expanded on in future work exploring factors that predict conversion of ideation to action. Given the PPV values we report [which are low but in line with the literature (Belsher et al., 2019)], it would be crucial that any interventions developed using similarly performing models be carefully designed in order to favor low-intensity interventions such as further assessment by a clinician over any restrictive or high-intensity interventions. Alternatively, future work could focus on improving the PPV and other metrics of these models.

There are several limitations to our current work. While using an interview-based census dataset allows for a large sample size in the general population, it does mean that there are no clinician-rated scales or independent verification of participant responses. Our use of deep learning, intended here as a proof of concept, provides a powerful technique that has the potential to match or outperform other methods as the dataset size increases (Alom et al., 2019). While deep learning is generally less easy to interpret than other machine learning techniques, our sensitivity analysis does allow some insight into the model parameters which could be further evaluated using classical statistics. This work is an initial proof of concept that could lead to a more comprehensive solution that would identify suicidal ideation and suicidal behaviours with increased reliability. While gradient boosting currently outperforms deep learning in this dataset, deep learning performed well and the sensitivity analysis renders it more interpretable, which in turn makes further study of deep learning models in larger datasets attractive. In addition, the sensitivity analysis may be useful in determining when a model which may have reasonably good metrics may have learned inappropriate relationships between features, potentially limiting generalizability to other datasets or to specific subsets of patients. Examining the results of a sensitivity analysis may uncover potentially spurious relationships learned by a model when the underlying data has an unexpected skew. Thus, when working with imbalanced datasets that require different sampling approaches [like the one in this paper], or when working with deep learning in datasets which are expected to contain known relationships between features, the sensitivity analysis may be a useful tool beyond model metrics in examining model quality and likely generalizability. A significant limitation to this current paper is the lack of an independent dataset for validation, notwithstanding our cross-validation approach in which test splits were designed to pose a harder problem than training splits by nature of the greater class imbalance in the test splits. Future work will need to test this model, or future successor models, on independent datasets to ensure generalizability.

We note that the data used in this paper had a significant breadth for each subject, with many different symptoms and socio-demographic and economic factors explored for each person included. However this comes at a cost of depth and specificity, with many features not necessarily containing all the information one might hope for. For example, our target feature—suicidal ideation—is a simple binary variable that does not include information about intensity, frequency or recurrence. In addition, it is not possible to verify the accuracy of each label of “yes” or “no” for suicidal ideation, with many “no”s potentially being from those who had ideations in the past and had forgotten, or who wished not to reveal ideations to a census agent. Despite this, the features identified by the model do seem reasonable when considered in the light of previous suicidal ideation literature, which speaks to the fact that the model seems to be picking up on realistic predictors. In addition, it must be remembered that this model is primarily a proof of concept, not a clinic-ready model, and will require further validation on different kinds of datasets prior to being ready for clinical testing.

One significant limitation of the model is that it identifies suicidal ideations rather than suicide attempt or completion risk, and as such its clinical utility is limited. For patients already being assessed by a mental health professional, or being interviewed for the purpose of determining SI, it is far simpler to simply ask them about lifetime or recent SI than to rely on or collect the data required to generate a model result. However, given that the data used to train this model was derived from the general population, it may have utility in the screening of patients in general practice, or *via* automatic review of electronic medical records. This could prompt clinicians to engage patients who they may not otherwise think to ask about SI, leading to more patients being referred to appropriate services as needed. In addition, the features identified in the larger 96 feature models may be helpful to those setting healthcare and social services priorities when considering interventions aimed at addressing or decreasing suicidal ideation. Prior to the deployment of any such model in the clinical setting further clinical research would be required.

Another relevant test for future models, trained on more examples of persons with suicidal ideation, would be to see if the model accurately recapitulates risk of suicidal ideation in specific strata of a risk-stratified population. In an effort to investigate how this set of models would perform in subgroups, we ran them three times each on different splits of data in order to see if model accuracy suffered when looking at subgroups within potentially relevant subject categories (for example, if the model had worse performance when used in only female subjects, or only people with a certain socioeconomic status). These results, detailed in Supplementary Table S9, demonstrate that the model performs well in all subgroups for which there was a reasonable amount of data. Future work should see models trained with and tested on larger proportions of patients from relevant subgroups in order to ensure that groups at risk of suicidal ideation, or traditionally underserved groups, are well represented in the model.

It is worth discussing the practical implementation of a tool for the identification of suicidal ideation in clinical practice, as this would bring both possible benefits and challenges. One possible implementation of this tool would be as an automated screening tool integrated into electronic medical records in emergency departments or outpatient clinics. Benefits—which would need to be verified in clinical studies—could include earlier and more accurate identification of suicidal ideation, which would lead to more patients being offered appropriate services, such as access to a therapist or to crisis resources. This in turn would hopefully lead to a reduction in the number of patients making suicide attempts or completing suicide, though this would depend on the efficacy of the offered interventions. Nonetheless, challenges and potential dangers exist. Models that identify suicidal ideation could be used by some clinicians to justify interventions such as forced hospitalization, which raises serious concerns about the effect of implementing such models on patient autonomy and clinician medico-legal risk. In addition, it is unclear what effect having an automated screening tool for suicidal ideation would have on clinician behavior. It might improve clinician awareness of the importance of screening for and offering support to patients with suicidal ideation. At the same time, since the model cannot currently identify all patients with SI or rule out all patients without SI, it may reinforce the habit of many clinicians to avoid asking about suicidal ideation, fostering an over-reliance on an imperfect system to screen for a potentially serious clinical phenomenon. Any implementation of such a screening system would require significant investment in the training of clinicians and should be accomplished in partnership with patient and clinician representatives.

## Data Availability

Publicly available datasets were analyzed in this study. This data can be found here: https://www150.statcan.gc.ca/n1/en/catalogue/82M0013X https://www23.statcan.gc.ca/imdb/p2SV.pl?Function=getSurvey&amp;Id=119789. Software Note: the Vulcan Platform can be accessed at https://github.com/Aifred-Health/Vulcan.

## Footnotes

1 A note on terminology: “feature” here refers to an input variable (i.e., one survey item).
