# Supplementary material for: Identification of Suicidal Ideation in the Canadian Community Health Survey—Mental Health Component Using Deep Learning
Source: Front Artif Intell. 2021 Jun 24;4:561528. doi: 10.3389/frai.2021.561528 (PMC8264793; doi:10.3389/frai.2021.561528)
Supplement: Supplementary file 1 [file Table1.docx]

**Appendix**

Table 1. Features retained in the 96-feature version of the lifetime suicidal ideation identification model

| Sociodemographic |
| --- |
| Employment status last week |
| Employment status in the past 12 months |
| Currently attending school, college, CEGEP, or university |
| Whether the respondent is an immigrant |
| Time in Canada since immigration |
| Highest level of education attained by any member of the respondent’s household |
| Lifestyle |
| Ever smoked cigarettes daily |
| Age when started smoking daily (daily smokers) |
| Number of cigarettes smoked per day (daily smokers) |
| Smoked 100 or more cigarette in their lifetime |
| Number of years since stopped smoking daily (former daily smokers) |
| Time since stopped smoking completely (former daily smokers) |
| Time since stopped smoking (never daily smokers) |
| Type of smoker (calculated)^^[[1]](#footnote-1)^^ |
| Age when first smoked a whole cigarette |
| Experienced alcohol abuse in their lifetime |
| Experienced alcohol abuse or dependence in their lifetime |
| Experienced drug abuse or dependence (including cannabis) in their lifetime |
| Self-perceived rating of ability to handle day-to-day demands |
| Self-perceived personal ability to deal with stress |
| Engaged in moderate or vigorous physical activity in the past week |
| Average number of hours of moderate or vigorous physical activity in the past week |
| Self-rated physical health |
| Perceived health |
| Satisfaction with life in general |
| Past Experiences or Trauma |
| Number of types of childhood maltreatment experienced |
| Had contact with the police in the past 12 months as a victim of a crime |
| Had contact with the police in the past 12 months for work |
| Had contact with the police in the past 12 months at a public information session |
| Psychiatric Diagnoses or Symptoms |
| Has a mood disorder^^[[2]](#footnote-2)^^ |
| Has a mood disorder: depression |
| Has a mood disorder other than depression, bipolar disorder, mania or dysthymia |
| Has ever consulted a medical doctor or other professional about symptoms of depression |
| Has ever been hospitalized overnight for symptoms of depression |
| Has an anxiety disorder such as phobia, panic disorder or obsessive-compulsive disorder |
| Has been treated for an emotional or mental health problem in the past 12 months |
| Consulted a doctor or another professional during the past year for symptoms of generalized anxiety disorder |
| Received professional treatment during the past year for symptoms of generalized anxiety disorder |
| Screened in general anxiety disorder^^[[3]](#footnote-3)^^ |
| Has had generalized anxiety disorder in the past 12 months |
| Duration of the longest episode of generalized anxiety disorder |
| Interference of generalized anxiety disorder on daily activities and responsibilities in the past 12 months^^[[4]](#footnote-4)^^ |
| Whether or not generalized anxiety disorder interfered with daily activities and responsibilities in the past 12 months |
| Experienced an episode of major depressive disorder in the past 12 months |
| Duration of the most recent major depressive episode |
| Interference of major depressive disorder on daily activities and responsibilities in the past 12 months^^[[5]](#footnote-5)^^ |
| Screened in mania^^[[6]](#footnote-6)^^ |
| Experienced bipolar II in their lifetime |
| Experienced mania in their lifetime |
| Experienced mania in the past 12 months |
| Age at occurence of most recent episode of manic feelings |
| Interference of mania on daily activities and responsibilities in the past 12 months^^[[7]](#footnote-7)^^ |
| Whether or not mania interfered with daily activities and responsibilities in the past 12 months^^[[8]](#footnote-8)^^ |
| Interference of hypomania on daily activities and responsibilities in the past 12 months^^[[9]](#footnote-9)^^ |
| Has used benzodiazepine medications in the past 2 days |
| Has used antidepressant medications in the past 2 days |
| Has used any medications for mental health, alcohol, or drugs in the past 2 days |
| Has used other health products for problems with emotions, mental health, alcohol, or drugs in the past 12 months |
| Total number of medications used in the past 2 days |
| World Health Organization (WHO) Disability Assessment Schedule (WHODAS) score |
| Amount of difficulty walking long distance in the past 30 days |
| Medical Comorbidities |
| Has chronic fatigue syndrome |
| Has bowel disorder, Crohn’s Disease or ulcerative colitis |
| Has heart disease |
| Has a chronic condition |
| Has diabetes |
| Has cancer |
| Has ever had cancer |
| Has migraine headaches |
| Has back problems excluding fibromyalgia and arthritis |
| Suffers from multiple chemical sensitivities |
| Ever diagnosed with high blood pressure |
| Has or was previously diagnosed with high blood pressure (derived from other items) |
| Degree of pain usually felt by the respondent and whether it prevents them from performing certain activities |
| Impact of negative opinions or unfair treatment on health care for physical health problems |
| Social Support |
| Has close relationships |
| Has people to count on in emergency |
| Has a trustworthy person to turn to for advice |
| Can count on people they know to help deal with their most important source of stress |
| Has people who admire their talents and abilities |
| Has people who enjoy the same activities |
| Has someone to talk to about important decisions |
| Has relationships that recognize competence and skill |
| Help received in the past 12 months |
| Amount of difficulty maintaining a friendship in the past 30 days |
| Has a friend who has ever been treated for an emotional or mental health problem |
| Family member has been treated for emotional or mental health in the past 12 months |
| Amount of time spent in an average week providing help to family member(s) |
| Extent to which the respondent's life is affected by family member(s)’s emotional, mental health, alcohol or drug problems |
| Help provided by respondent to family member(s) by providing company or emotional support |
| Help provided by respondent to family member(s) by spending time doing other things^^[[10]](#footnote-10)^^ related to their problems |
| Family member(s)’s problems cause respondent worry, anxiety or depression |
| Family member(s)’s problems cause respondent embarrassment |
| How often the respondent felt that others did things that were thoughtless or inconsiderate in the past month |
| How often the respondent felt that others were critical of their behaviour in the past month |
| Impact of negative opinions or unfair treatment on romantic life |
| Other |
| Interview by telephone or in person |

Table 2. Features retained in the 21-feature version of the lifetime suicidal ideation identification model.

Results of sensitivity analysis expressed as total numbers and ratios are presented in the middle columns. These express how many people would be classified as having suicidal ideation if all respondents tested gave answers at one or another extreme within the value range for a given question. For example, in row one, if all tested participants answered that they had lifetime drug abuse or cannabis dependence, then there would be 9,055 positive classifications of suicidal ideation, and this would drop to 4,538 if all samples had never had drug abuse or dependence. The third column describes the ratio of these two numbers. The rightmost column provides the true distributions in the training set. Note that the train set distribution and model sensitivity analysis were performed on two different train sets (two separate runs), thus, some train set distributions containing very few feature value examples may not have sensitivities that line up intuitively.

| **Feature Description** | **Model Sensitivity: Possible Response (number of samples classified as having suicidal ideation)** | **Model Sensitivity: Ratios** | **Train Set: Distribution** |
| --- | --- | --- | --- |
| Drug abuse or dependence (including cannabis): life (Derived) | Yes (9,055),  No (4,538) | Yes vs. No  (1.995 : 1) | Yes  (SI : 422, Not SI : 108),  No  (SI : 1577, Not SI : 1905) |
| Number of times before age 16 was physically attacked (kicked/bitten/punched/choked/burned/other) | 6 to 10 Times (18501),  Never (4349) | 6 to 10 Times vs. Never  (4.254 : 1) | 6 to 10 Times  (SI : 47, Not SI : 12),  Never  (1361, Not SI : 1712) |
| Full-time or part-time working status (Derived) | Part-time (5985),  Full-time (4947) | Part-time vs. Full-time  (1.210 : 1) | Part-time  (SI : 269, Not SI : 229)  Full-time  (SI : 939, Not SI : 863) |
| Has people who enjoy same activities | Strongly Disagree (9621), Strongly Agree (4681) | Strongly Disagree vs. Strongly Agree  (2.055 : 1) | Strongly Disagree  (SI : 35, Not SI : 15)  Strongly Agree  (SI : 1007, Not SI 1177) |
| Number of years stopping smoking completely (grouped) | 6 To 10 Years (6919),  11 Or More Years (4394) | 6 To 10 Years vs. 11 Or More Years  (1.575 : 1) | 6 To 10 Years  (SI : 106, Not SI : 70)  11 Or More Years  (SI : 307, Not SI : 368) |
| Number of times before age 16 was pushed, grabbed, shoved or thrown at by adult | More Than 10 Times (11910),  Never (4133) | More Than 10 Times vs.  Never  (2.882 : 1) | More Than 10 Times  (SI : 261, Not SI : 70),  Never  (SI : 1107, Not SI : 1538) |
| Has personally ever received treatment for emotional or mental health problem | Yes (14145),  No (3399) | Yes vs. No  (4.162 : 1) | Yes  (SI : 934, Not SI : 277)  No  (SI : 1097, Not SI : 1754) |
| Age at occurrence of most recent episode of manic feelings (Derived) | 75 to 79 years (19774)  Less than 15 years (1) | 75 to 79 years vs. Less than 15 years  (19774 : 1) | 75 to 79 years  (SI : 1, Not SI : 0)  Less than 15 years  (SI : 0, Not SI : 0) |
| Alcohol abuse: Algorithm: life (Derived) | Yes (6150),  No (4526) | Yes vs. No  (1.359 : 1) | Yes  (SI : 497, Not SI : 295),  No  (SI : 1507, Not SI : 1721) |
| Has a strong emotional bond with at least one other person | Disagree (6243),  Strongly Disagree (4355) | Disagree vs.  Strongly Disagree  (1.434 : 1) | Disagree  (SI : 79, Not SI : 52),  Strongly Disagree  (SI : 19, Not SI : 6) |
| Has bowel disorder, Crohn's Disease or ulcerative colitis | Yes (5624),  No (4834) | Yes vs. No  (1.163 : 1) | Yes  (SI : 171, Not SI : 119),  No  (SI : 1864, Not SI : 1915) |
| Generalized anxiety disorder: algorithm: life (Derived) | Yes (9432),  No (4617) | Yes vs. No  (2.043 : 1) | Yes  (SI : 417, Not SI : 136),  No  (SI : 1592, Not SI : 1887) |
| Amount of difficulty in standing for long periods in past 30 days (grouped) | Moderate (6563),  None (4632) | Moderate vs. None  (1.419 : 1) | Moderate  (SI : 212, Not SI : 112),  None  (SI : 1460, Not SI : 1648) |
| Belong to a group or people who share attitudes and beliefs | Strongly Disagree (11199),  Strongly Agree (4277) | Strongly Disagree vs.  Strongly Agree  (2.618 : 1) | Strongly Disagree  (SI : 51, Not SI : 16),  Strongly Agree  (SI : 795, Not SI : 1070) |
| Has migraine headaches | Yes (8108),  No (4524) | Yes vs. No  (1.792 : 1) | Yes  (SI : 355, Not SI : 181),  No  (SI : 1680, Not SI 1853) |
| Has other long-term physical or mental health condition (grouped) | Yes (6511),  No (4601) | Yes vs. No  (1.415 : 1) | Yes  (SI : 493, Not SI : 322),  No  (SI : 1543, Not SI : 1710) |
| Has friend who has ever been treated for emotional or mental health problem | Yes (6212),  No (4286) | Yes vs. No  (1.449 : 1) | Yes  (SI : 808, Not SI : 490),  No  (SI : 1186, Not SI : 1508) |
| Has high blood pressure | No (5018),  Yes (4322) | No vs. Yes  (1.161 : 1) | No  (SI : 1649, Not SI : 1556)  Yes  (SI : 384, Not SI : 473 ) |
| Has relationships that recognize competence and skill | Disagree (7092),  Agree (4352) | Disagree vs. Agree  (1.660 : 1) | Disagree  (SI : 109, Not SI : 58)  Agree  (SI : 927, Not SI : 826) |
| Self-perceived rating of ability to handle day-to-day demands | Poor (7575),  Excellent (4215) | Poor vs. Excellent  (1.797 : 1) | Poor  (SI : 34, Not SI : 15)  Excellent  (SI : 336, Not SI : 447) |
| Generalized anxiety disorder: algorithm: 12 months (Derived) | No (4876),  Yes (4822) | No vs. Yes  (1.011) | No  (SI : 1889, Not SI : 1990),  Yes  (SI : 122, Not SI : 33) |

Table 3. Features retained in the 96-feature version of the last 12 months suicidal ideation identification model.

| **Sociodemographic** |
| --- |
| Age |
| Sex |
| Marital status |
| Employment status last week |
| Employment status in the past 12 months |
| Currently attending school, college, CEGEP, or university |
| Whether the respondent is an immigrant |
| Highest level of education attained by any member of household |
| **Lifestyle** |
| Age when first smoked a whole cigarette |
| Age when started smoking daily (daily smokers) |
| Ever smoked cigarettes daily |
| Age when started smoking daily (former daily smokers) |
| Number of cigarettes smoked per day (occasional smokers) |
| Number of years since stopped smoking daily (former daily smokers) |
| Number of years since stopped smoking (never daily smokers) |
| Ever smoked a whole cigarette |
| Number of days in the past month when smoked one cigarette or more (occasional smoker) |
| Type of smoker |
| Type of smoker (calculated)^^[[11]](#footnote-11)^^ |
| Number of years smoked |
| Experienced alcohol abuse or dependence in their lifetime |
| Experienced drug abuse or dependence (including cannabis) in their lifetime |
| Experienced drug abuse or dependence (excluding cannabis) in their lifetime |
| World Health Organization (WHO) Disability Assessment Schedule (WHODAS) score |
| Extent to which religious or spiritual values provide strength to face everyday difficulties |
| Self-rated physical health |
| **Past Experiences or Trauma** |
| Had contact with the police in the past 12 months as a victim of a crime |
| Had contact with the police in the past 12 months as a witness to a crime |
| Had contact with the police in the past 12 months for other^^[[12]](#footnote-12)^^ reason |
| Number of times before age 16 was slapped, hit or spanked by adult |
| Number of times before age 16 experienced forced sexual touching by an adult |
| **Psychiatric Diagnoses or Symptoms** |
| Has post-traumatic stress disorder |
| Has a mood disorder^^[[13]](#footnote-13)^^ |
| Whether or not the major depressive episode interfered significantly in daily activities and responsibilities in the past 12 months |
| Interference of major depressive disorder on daily activities and responsibilities in the past 12 months^^[[14]](#footnote-14)^^ |
| Has an anxiety disorder such as phobia, panic disorder or obsessive-compulsive disorder |
| Screened in generalized anxiety disorder^^[[15]](#footnote-15)^^ |
| Has had generalized anxiety disorder in the past 12 months |
| Age at occurence of most recent episode of generalized anxiety disorder |
| Experienced generalized anxiety disorder in their lifetime |
| Received professional treatment during the past year for symptoms of generalized anxiety disorder |
| Whether or not generalized anxiety disorder interfered significantly in daily activities and responsibilities in the past 12 months |
| Age at occurence of most recent episode of manic feelings |
| Duration of manic feelings |
| Whether or not mania interfered significantly in daily activities and responsibilities in the past 12 months |
| Interference of mania on daily activities and responsibilities in the past 12 months^^[[16]](#footnote-16)^^ |
| Interference of hypomania on daily activities and responsibilities in the past 12 months^^[[17]](#footnote-17)^^ |
| Score on the personal impact scale of mental health experiences |
| Help received in the past 12 months^^[[18]](#footnote-18)^^ |
| Consulted a psychiatrist for emotional/mental health/alcohol/drug problems in the past 12 months |
| Consulted a family doctor or general practitioner for emotional/mental health alcohol/drug problems in the past 12 months |
| Used medications for problems with emotions, mental health, alcohol, or drugs in the past 12 months |
| Used medications other than antidepressants, benzodiazepines and antipsychotics in the past 2 days |
| Extent to which the respondent has been emotionally affected by their own health problems in the past 30 days |
| Self-perceived personal ability to deal with stress |
| Amount of difficulty concentrating on tasks in the past 30 days |
| Amount of difficulty in day-to-day work or school activities in the past 30 days |
| Amount of difficulty taking care of household responsibilities in the past 30 days |
| Amount of difficulty joining in community activities in the past 30 days |
| Amount of difficulty dealing with people the respondent does not know in the past 30 days |
| Amount of difficulty standing for long periods in the past 30 days |
| Extent to which the above difficulties interfered with the respondent’s life |
| **Medical Comorbidities** |
| Has cancer |
| Has diabetes |
| Has high blood pressure |
| Has or was previously diagnosed with high blood pressure |
| Suffers from the effects of a stroke |
| Has a bowel disorder, Crohn's Disease or ulcerative colitis |
| Suffers from multiple chemical sensitivities |
| Degree of pain usually felt by the respondent and whether it prevents them from performing certain activities |
| Opinion of own weight |
| Body mass index (BMI) |
| Body mass index (BMI) classification (underweight, normal weight, overweight, or obese) |
| **Social Support** |
| Has close relationships |
| Has relationships that recognize competence and skill |
| Has a trustworthy person to turn for advice |
| Has someone to talk to about important decisions |
| Can count on people they know to help deal with their most important source of stress |
| Any family member has emotional, mental health, alcohol or drug problems |
| Extent to which the respondent's life is affected by family member(s)’s emotional, mental health, alcohol or drug problems |
| Help provided by the respondent to their family member(s) with practical things |
| Help provided by respondent to family member(s) by providing company or emotional support |
| Help provided by the respondent to their family member(s) by spending time doing other things related to their problems |
| Amount of time spent in average week providing help to family member(s) |
| Has a friend who has been treated for an emotional or mental health problem in the past 12 months |
| Has a friend who has ever been treated for an emotional or mental health problem |
| Regular contact with people is detrimental to their wellbeing due to discomfort and stress |
| Family member(‘s’) problems cause the respondent worry, anxiety or depression |
| Family member(‘s’) problems cause the respondent embarrassment |
| Impact of negative opinions or unfair treatment on family relationships^^[[19]](#footnote-19)^^ |
| Impact of negative opinions or unfair treatment on romantic life^^[[20]](#footnote-20)^^ |
| Impact of negative opinions or unfair treatment on housing situation^^[[21]](#footnote-21)^^ |
| How often the respondent felt that others were critical of their behaviour in the past month |
| How often the respondent felt that others acted angry or upset with them in the past month |
| Amount of negative social interactions |
| **Other** |
| Answers were affected by the presence of another person |

Table 4. Features retained in the 21-feature version of the last 12 months suicidal ideation identification model.

Results of sensitivity analysis expressed as total numbers and ratios are presented in the two middle columns. Train set distribution shown in right most column. Note that the train set distribution and model sensitivity analysis were performed on two different train sets (two separate runs), thus, some train set distributions containing very few feature value examples may not have sensitivities that line up intuitively.

| **Feature Description** | **Model Sensitivity: Response (number of samples classified as SI)** | **Model Sensitivity: Ratios** | **Train Set: Distribution** |
| --- | --- | --- | --- |
| Screened in Depression (Derived) | Yes (582),  No (316) | Yes vs. No  (1.842 : 1) | Yes  (SI : 774, Not SI : 738),  No  (SI : 54, Not SI : 106) |
| Currently pregnant | No (560),  Yes (297) | No vs. Yes  (1.886 : 1) | No  (SI : 288, Not SI : 258)  Yes  (SI : 7, Not SI : 8) |
| Has asthma | Yes (673),  No (531) | Yes vs. No  (1.267 : 1) | Yes  (SI : 139, Not SI : 125),  No  (SI : 687, Not SI : 718) |
| Has Attention Deficit Disorder | Yes (893),  No (534) | Yes vs. No  (1.672 : 1) | Yes  (SI : 88, Not SI : 58),  No  (SI : 737, Not SI : 786) |
| Chronicity of distress/ impairment scale for past month (Derived) | Somewhat less than usual (1027),  Never had any (354) | Somewhat less than usual vs. Never had any  (2.901 : 1) | Somewhat less than usual  (SI : 49, Not SI : 41),  Never had any  (SI : 17, Not SI : 53) |
| Mania: 2002 Algorithm: 12 months (Derived) | Yes (1027),  No (550) | Yes vs. No  (1.867 : 1) | Yes  (SI : 91, Not SI : 21),  No  (SI : 719, Not SI : 821), |
| Mania: Interference: Mean: 12 months (Derived) | Score 7 (1608),  Score 6 (422) | Score 7 vs. Score 6  (3.810 : 1) | Score 7  (SI : 5, Not SI : 2),  Score 6  (SI : 1, Not SI : 1) |
| How long ago stopped smoking (former daily smokers, grouped) | Less than 1 year (692),  1 to less than 2 years (242) | Less than 1 year vs. 1 to less than 2 years  (2.860 : 1) | Less than 1 year  (SI : 39, Not SI : 35),  1 to less than 2 years  (SI : 12, Not SI : 13) |
| Alcohol abuse or dependence: Algorithm: life (Derived) | Yes (678),  No (510) | Yes vs. No  (1.329 : 1) | Yes  (SI : 318, Not SI : 291),  No  (SI : 498, Not SI : 540) |
| Drug abuse or dependence (excluding cannabis): life (Derived) | No (550),  Yes (480) | No vs. Yes  (1.146 : 1) | No  (SI : 148, Not SI : 130),  Yes  (SI : 671, Not SI : 702) |
| Drug abuse or dependence (including cannabis): life (Derived) | Yes (613),  No (525) | Yes vs. No  (1.168 : 1) | Yes  (SI : 233, Not SI : 183),  No  (SI : 582, Not SI : 649) |
| Amount of difficulty walking long distance in past 30 days (grouped) | Extreme (894),  None (474) | Extreme vs. None  (1.886 : 1) | Extreme  (SI : 90, Not SI : 69),  None  (SI : 544, Not SI : 605) |
| Has personally ever received treatment for emotional or mental health problem | Yes (629),  No (468) | Yes vs. No  (1.344 : 1) | Yes  (SI : 517, Not SI : 471),  None  (SI : 371, Not SI : 309) |
| Help provided by respondent to family member(s) by providing company or emotional support | Yes (568),  No (517) | Yes vs. No  (1.099 : 1) | Yes  (SI : 130, Not SI : 111),  None  (SI : 109, Not SI : 107) |
| Has a trustworthy person to turn to for advice | Strongly Disagree (1612)  Strongly Agree (389) | Strongly Disagree vs Strongly Agree  (4.144 : 1) | Strongly Disagree  (SI : 25, Not SI : 5),  Strongly Agree  (SI : 383, Not SI : 535) |
| Negative social interactions scale (Derived) | Score of 6 or more (1079),  Score of 5 or less (489) | Score of 6 or more vs. Score of 5 or less  (2.206 : 1) | Score of 6 or more  (SI : 203, Not SI : 101),  Score of 5 or less  (SI : 531, Not SI : 670) |
| Number of times before age 16 was slapped, hit or spanked by an adult | Never (546),  More than 10 times (448) | Never vs. More than 10 times  (1.219 : 1) | Never  (SI : 244, Not SI : 305),  More than 10 times  (SI : 192, Not SI : 188) |
| Importance of religious or spiritual values in daily life | Somewhat Important (663),  Very Important (486) | Somewhat Important vs. Very Important  (1.097 : 1) | Somewhat Important  (SI : 225, Not SI : 210),  Very Important  (SI : 240, Not SI : 286) |
| Full-time or part-time working status (Derived) | Part time (646),  Full time (430) | Part time vs. Full time  (1.502 : 1) | Part time  (SI : 116, Not SI : 105),  Full time  (SI : 300, Not SI : 387) |
| Currently attending school, college, CEGEP, or university | No (584),  Yes (379) | No vs. Yes  (1.541 : 1) | No  (SI : 693, Not SI : 729),  Yes  (SI : 131, Not SI : 111) |
| Has arthritis | No (604),  Yes (451) | No vs. Yes  (1.339 : 1) | No  (SI : 630, Not SI : 629)  Yes  (SI : 196, Not SI : 213) |

Table 5. Lifetime and last-12-months suicidal ideation identification model metrics, including comparison between random forest baseline model and deep learning (cross entropy loss function) results.

* DL = Deep Learning; RF = Random Forest; GBT = Gradient-Boosted Tree

| **Lifetime** | **Number of Features** | **Loss Function** | **Sensitivity** | **Specificity** | **AUC** | **PPV** | **NPV** |
| --- | --- | --- | --- | --- | --- | --- | --- |
|  | 96 | DL | 0.6723 | 0.7630 | 0.7890 | 0.0316 | 0.9951 |
|  | 96 | RF | 0.6564 | 0.7643 | 0.7809 | 0.0300 | 0.9930 |
|  | 96 | GBT | 0.7453 | 0.7482 | 0.8291 | 0.0320 | 1.000 |
|  | 21 | DL | 0.6214 | 0.7737 | 0.7681 | 0.0308 | 0.9944 |
|  | 21 | RF | 0.6435 | 0.7298 | 0.7555 | 0.0280 | 0.9940 |
|  | 21 | GBT | 0.7271 | 0.7346 | 0.8142 | 0.0300 | 1.000 |
| **Last 12 Months** | **Number of Features** | **Loss Function** | **Sensitivity** | **Specificity** | **AUC** | **PPV** | **NPV** |
|  | 96 | DL | 0.5972 | 0.7050 | 0.7081 | 0.1293 | 0.9665 |
|  | 96 | RF | 0.6158 | 0.7561 | 0.7341 | 0.1350 | 0.9710 |
|  | 96 | GBT | 0.6752 | 0.7803 | 0.8016 | 0.1580 | 0.9740 |
|  | 21 | DL | 0.5878 | 0.6702 | 0.6798 | 0.074 | 0.9642 |
|  | 21 | RF | 0.6485 | 0.7297 | 0.7395 | 0.1280 | 0.9710 |
|  | 21 | GBT | 0.6782 | 0.7614 | 0.7926 | 0.1450 | 0.9760 |

**Supplementary Materials**

Table 6. Sizes of datasets and distribution of data.

|  | Lifetime Suicidal Ideation | Last-12-Months Suicidal Ideation |
| --- | --- | --- |
| Total number of examples | 23,859 | 3,441 |
| Number of examples that answered ‘yes’ | 2,262 | 929 |
| Number of examples that answered ‘no’ | 21,597 | 2,512 |
| Number of males | 10,871 | 1,437 |
| Number of females | 12,988 | 2,004 |
| 60 TO 64 YEARS | 2,104 | 280 |
| 55 TO 59 YEARS | 2,097 | 376 |
| 15 TO 19 YEARS | 1,932 | 291 |
| 20 TO 24 YEARS | 1,886 | 330 |
| 65 TO 69 YEARS | 1,844 | 209 |
| 50 TO 54 YEARS | 1,821 | 329 |
| 30 TO 34 YEARS | 1,772 | 279 |
| 35 TO 39 YEARS | 1,616 | 279 |
| 40 TO 44 YEARS | 1,585 | 282 |
| 45 TO 49 YEARS | 1,571 | 254 |
| 80 YEARS OR MORE | 1,560 | 72 |
| 25 TO 29 YEARS | 1,521 | 279 |
| 70 TO 74 YEARS | 1,388 | 114 |
| 75 TO 79 YEARS | 1,162 | 67 |

Table 7. Sensitive cost function experiment results

The rows in Table 7 have the sensitive cost function that indicate an experimental approach where instead of using the simple cross entropy loss function, we added an extra penalty to the false negatives, in the hope of improving the number of false negatives. As can be seen, the sensitivity did improve slightly for the 96 and 21 feature lifetime datasets. However, it actually decreased the sensitivity in both data subsets for the last 12 months classification . This could have been because the number of samples in the last 12 months dataset is extremely small, and thus the sensitive cost function did not have the desired effect of being exposed to suboptimal levels of data variation.

| **Number of Features** | **Sensitivity** | **Specificity** | **AUC** | **PPV** | **NPV** |
| --- | --- | --- | --- | --- | --- |
| Lifetime - 96 | 0.7140 | 0.7436 | 0.7995 | 0.03120 | 0.9955 |
| Lifetime - 21 | 0.6369 | 0.7189 | 0.7486 | 0.02560 | 0.9941 |
| Last 12 months - 96 | 0.6603 | 0.7155 | 0.7494 | 0.1138 | 0.9744 |
| Last 12 months - 21 | 0.5909 | 0.6879 | 0.6884 | 0.0946 | 0.9683 |

**Methods Addendum**

Variance Thresholding

Variance thresholding, a method which removes columns (i.e. features) if they do not vary sufficiently across the patient samples depending on the threshold given, was attempted but discontinued as it seemed to adversely affect the predictive power of the results. We presume this may have been due to the extreme imbalance in the dataset, where columns removed via this method may have in fact been the determining features that helped distinguish the difference between having suicidal ideation and not having them.

Table 8. Class imbalance experiments

Oversampling was experimented with by repeating the small positive class a few times in the train set in order to have more examples from the negative class for training. Specifically, we copied 80% of the positive class three times and equated that number to the number of negative samples chosen for the train set. The rest of the data was used for the validation set. Both techniques have very similar results, and thus, we chose to use undersampling technique as our main since it is less sensitive to feature reduction than the oversampling technique.

|  | **Number of Features** | **Undersampling 10-fold cross validated AUC** | **Oversampling 10-fold cross validated AUC** | **Undersampling 10-fold Gradient Boosting AUC** |
| --- | --- | --- | --- | --- |
| **Lifetime** | 96 | 0.7983 | 0.8025 | 0.8123 |
|  | 21 | 0.7550 | 0.7304 | 0.760 |
| **Last 12 Months** | 96 | 0.7611 | 0.7886 | 0.789 |
|  | 21 | 0.6913 | 0.6940 | 0.710 |

Table 9. Stratified results

This table documents the average of three runs of each of the four models. The feature name column details the feature used to define the subgroup and the feature description column details the different levels present within each feature. The average of the three runs then details the number of patients within each level of a feature and the AUC for that level of the feature. Note that when an N/A is shown in an AUC cell, this is because all of the samples in one level of a subgroup provided the same response (i.e. all “yes” or all “no”), precluding the calculation of an AUC. These results demonstrate that the models performed well even in subsets of the population which may be relevant for identifying SI, for example even when comparing men to women.

| **Model** | **Feature name** | **Feature Description** | **AUC (average across 3 runs)** | **Number of Patients (average across 3 runs)** |
| --- | --- | --- | --- | --- |
| 96 year | Highest level of education attained by respondent: 4 levels (Derived) | NOT STATED | **0.930** | **12** |
|  |  | POST-SEC. GRAD. | **0.770** | **1027** |
|  |  | SOME POST-SEC. | **0.770** | **143** |
|  |  | SECONDARY GRAD. | **0.747** | **278** |
|  |  | < THAN SECONDARY | **0.697** | **309** |
|  | Sex | MALE | **0.760** | **722** |
|  |  | FEMALE | **0.777** | **1047** |
|  | Total household income from all sources (Derived/Grouped) | NO INCOME OR LESS THAN $20,000 | **0.825** | **200** |
|  |  | $20,000-$39,999 | **0.720** | **361** |
|  |  | $40,000-$59,999 | **0.770** | **368** |
|  |  | $60,000-$79,999 | **0.743** | **294** |
|  |  | $80,000 OR MORE | **0.815** | **546** |
|  |  | NOT STATED | **N/A** | **1** |
| 21 year | Highest level of education attained by respondent: 4 levels (Derived) | NOT STATED | **0.860** | **12** |
|  |  | POST-SEC. GRAD. | **0.713** | **1030** |
|  |  | SOME POST-SEC. | **0.687** | **160** |
|  |  | SECONDARY GRAD. | **0.697** | **270** |
|  |  | < THAN SECONDARY | **0.643** | **296** |
|  | Sex | MALE | **0.710** | **826** |
|  |  | FEMALE | **0.717** | **1040** |
|  | Total household income from all sources (Derived/Grouped) | NO INCOME OR LESS THAN $20,000 | **0.637** | **195** |
|  |  | $20,000-$39,999 | **0.693** | **342** |
|  |  | $40,000-$59,999 | **0.710** | **372** |
|  |  | $60,000-$79,999 | **0.710** | **295** |
|  |  | $80,000 OR MORE | **0.710** | **564** |
|  |  | NOT STATED | **1.000** | **1** |
| 96 lifetime | Highest level of education attained by respondent: 4 levels (Derived) | NOT STATED | **0.755** | **76** |
|  |  | POST-SEC. GRAD. | **0.810** | **11035** |
|  |  | SOME POST-SEC. | **0.763** | **1236** |
|  |  | SECONDARY GRAD. | **0.807** | **3180** |
|  |  | < THAN SECONDARY | **0.803** | **4261** |
|  | Sex | MALE | **0.820** | **9036** |
|  |  | FEMALE | **0.793** | **10752** |
|  | Total household income from all sources (Derived/Grouped) | NO INCOME OR LESS THAN $20,000 | **0.853** | **1219** |
|  |  | $20,000-$39,999 | **0.780** | **3380** |
|  |  | $40,000-$59,999 | **0.800** | **4202** |
|  |  | $60,000-$79,999 | **0.797** | **3312** |
|  |  | $80,000 OR MORE | **0.807** | **7663** |
|  |  | NOT STATED | **N/A** | **11** |
| 21 lifetime | Highest level of education attained by respondent: 4 levels (Derived) | NOT STATED | **0.550** | **74** |
|  |  | POST-SEC. GRAD. | **0.623** | **11050** |
|  |  | SOME POST-SEC. | **0.697** | **1246** |
|  |  | SECONDARY GRAD. | **0.713** | **3157** |
|  |  | < THAN SECONDARY | **0.690** | **4261** |
|  | Sex | MALE | **0.720** | **9605** |
|  |  | FEMALE | **0.710** | **10774** |
|  | Total household income from all sources (Derived/Grouped) | NO INCOME OR LESS THAN $20,000 | **0.685** | **1213** |
|  |  | $20,000-$39,999 | **0.703** | **3387** |
|  |  | $40,000-$59,999 | **0.687** | **4197** |
|  |  | $60,000-$79,999 | **0.763** | **3303** |
|  |  | $80,000 OR MORE | **0.690** | **7677** |
|  |  | NOT STATED | **N/A** | **11** |

Figure 1. Flow of data through our training and inference system broken into three phases, 1) Data Processing – reduce dataset features using expert reduction & first layer weight analyses, 2) Model Training & Testing – 10-fold cross validation using undersampling of the ‘no’ class and training a neural network, and 3) Sensitivity Analysis – discovering feature directionality for our 21 feature trained models.


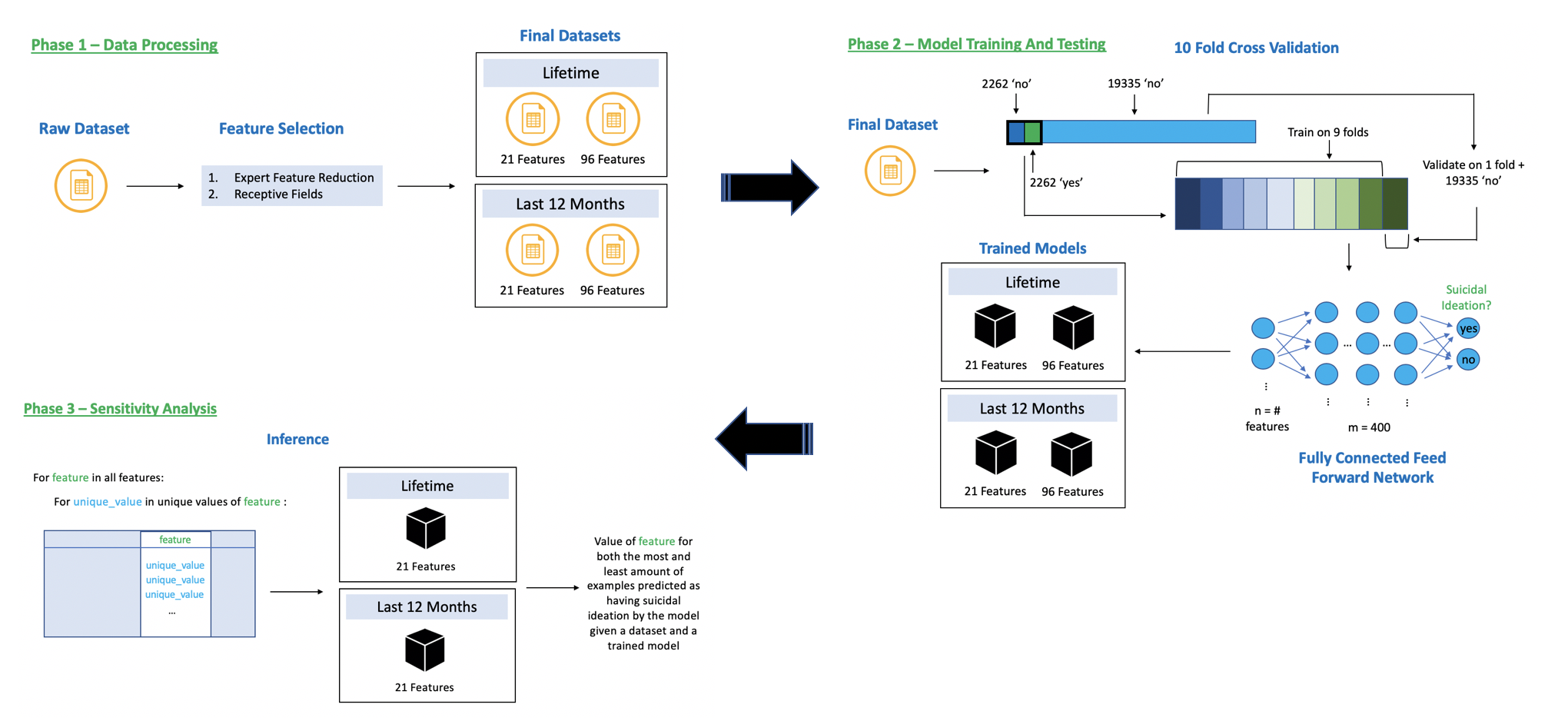


Figure 2. Example of receptive fields feature reduction technique for lifetime dataset. 1) Model Training – train model with all 196 features remaining after expert feature reduction using lifetime ideation as target. 2) Feature Removal – Remove features that the model learned the lowest weights for until you have X features left. (X = 21 or 96 in our case)


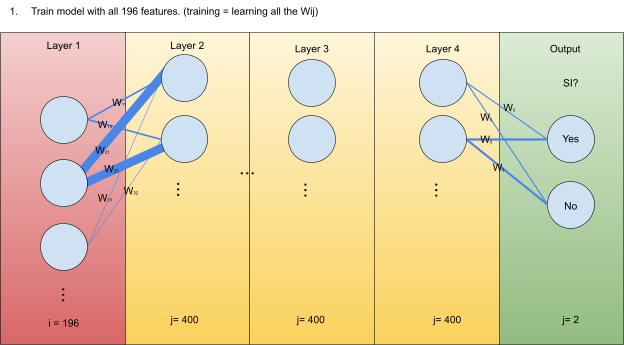


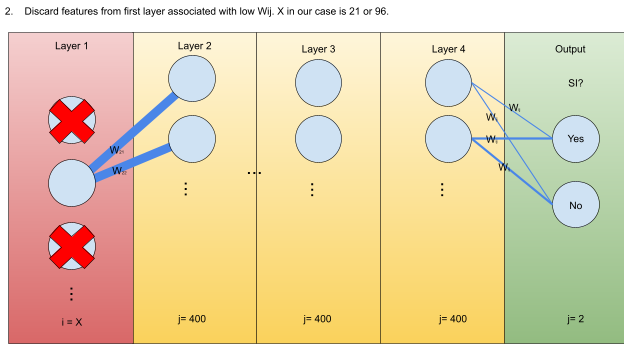


Figure 3. 10-fold cross validated performance of all four models across different neural network architectures.


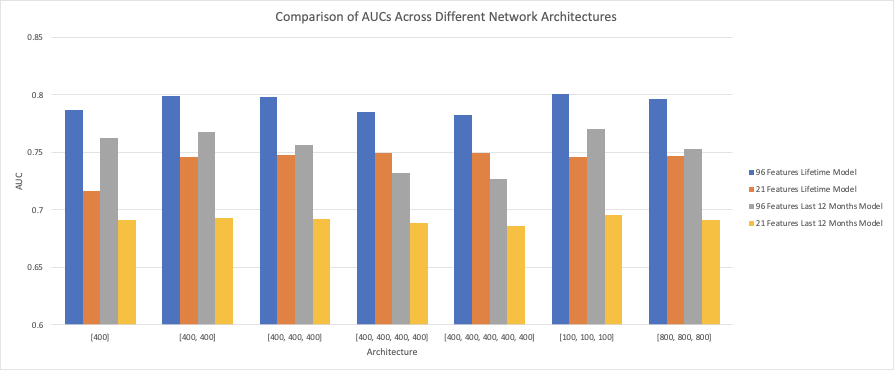


1. This question was computed from the results of other questions about smoking: if the respondent has smoked 100 or more cigarettes during their lifetime, if the respondent has ever smoked a whole cigarette, what type of smoker the respondent is, if the respondent has ever smoked cigarettes daily. This calculation produces a 1 (daily smoker) to 6 (never smoked) score. [↑](#footnote-ref-1)
2. Depression, bipolar disorder, mania or dysthymia [↑](#footnote-ref-2)
3. This question was computed from the results of other screening questions for generalized anxiety disorder: if the respondent ever had a time when they worried much more about things than other people with the same problems; if the respondent ever had a time when they were much more nervous or anxious than most other people with the same problems; if the respondent ever had a period of 6 months or more when they were anxious or worried most days. [↑](#footnote-ref-3)
4. On a scale of 0 (low interference) to 10 (high interference) [↑](#footnote-ref-4)
5. On a scale of 0 (low interference) to 10 (high interference) [↑](#footnote-ref-5)
6. This question was computed from the results of other screening questions for mania: if the respondent ever had a manialike period lasting several days or longer; if the respondent ever had a period of days when they were so irritable that they either started arguments, shouted at people or hit people. [↑](#footnote-ref-6)
7. On a scale of 0 (low interference) to 10 (high interference) [↑](#footnote-ref-7)
8. On a scale of 0 (low interference) to 10 (high interference) [↑](#footnote-ref-8)
9. On a scale of 0 (low interference) to 10 (high interference) [↑](#footnote-ref-9)
10. All things other than providing practical support, company or emotional support [↑](#footnote-ref-10)
11. This question was computed from the results of other questions about smoking: if the respondent has smoked 100 or more cigarettes during their lifetime, if the respondent has ever smoked a whole cigarette, what type of smoker the respondent is, if the respondent has ever smoked cigarettes daily. This calculation produces a 1 (daily smoker) to 6 (never smoked) score. [↑](#footnote-ref-11)
12. Reason other than being the victim of a crime, a witness to a crime, for work, for a public information session, due to a traffic violation or accident, or due to a family member’s emotional, mental health, alcohol or drug problems [↑](#footnote-ref-12)
13. Depression, bipolar disorder, mania or dysthymia [↑](#footnote-ref-13)
14. On a scale of 0 (low interference) to 10 (high interference) [↑](#footnote-ref-14)
15. This question was computed from the results of other screening questions for generalized anxiety disorder: if the respondent ever had a time when they worried much more about things than other people with the same problems; if the respondent ever had a time when they were much more nervous or anxious than most other people with the same problems; if the respondent ever had a period of 6 months or more when they were anxious or worried most days. [↑](#footnote-ref-15)
16. On a scale of 0 (low interference) to 10 (high interference) [↑](#footnote-ref-16)
17. On a scale of 0 (low interference) to 10 (high interference) [↑](#footnote-ref-17)
18. Based on 5 answers about types of help: a) information about mental health problems, their treatments or available services; b) medication; c) counselling or therapy; d) other; e) none. [↑](#footnote-ref-18)
19. On a scale of 0 (has not been affected) to 10 (has been severely affected) [↑](#footnote-ref-19)
20. On a scale of 0 (has not been affected) to 10 (has been severely affected) [↑](#footnote-ref-20)
21. On a scale of 0 (has not been affected) to 10 (has been severely affected) [↑](#footnote-ref-21)
